# Supplementary figures and images for: Purkinje cell development and degeneration in the spastic Han-Wistar rat model of ataxia
Source: bioRxiv. 2025 Sep 23:2025.09.23.677887. Preprint. [Version 1] doi: 10.1101/2025.09.23.677887 (PMC12485880; doi:10.1101/2025.09.23.677887)

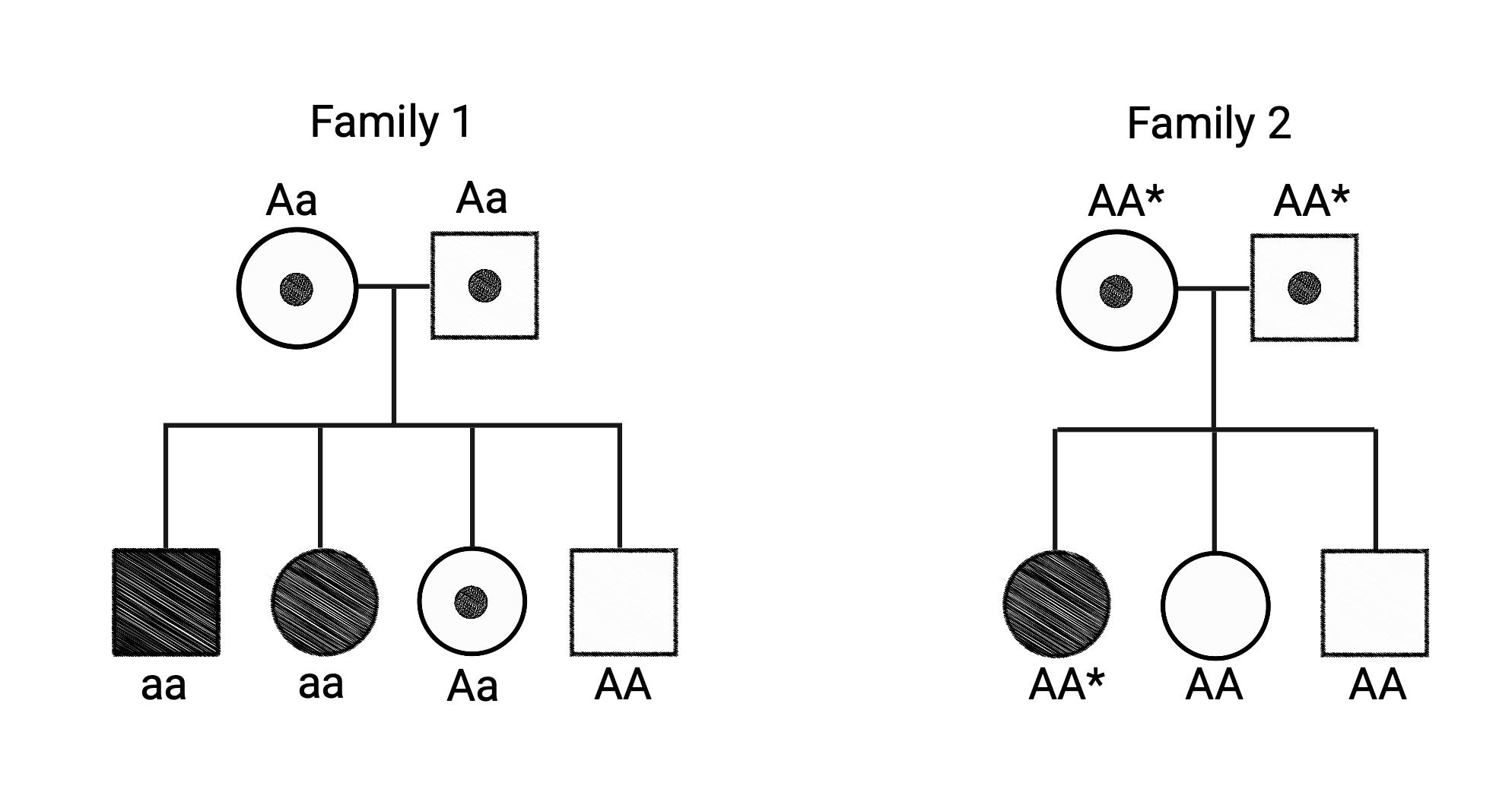

Supplement: Supplement 1 — Supplementary Figure 1. Pedigrees for candidate gene validation. In Family 1, whole-genome sequencing was performed on the mother, father, and one affected offspring. Sanger sequencing of the Sbf2 gene was performed for all individuals in both families. [file media-1.jpg]
